# Diagnostic Value of ^18^F-NOTA-FAPI PET/CT in a Rat Model of Radiation-Induced Lung Damage

**DOI:** 10.3389/fonc.2022.879281

**Published:** 2022-06-02

**Authors:** Xueting Qin, Shijie Wang, Xiaoli Liu, Jinghao Duan, Kai Cheng, Zhengshuai Mu, Jing Jia, Yuchun Wei, Shuanghu Yuan

**Affiliations:** ^1^ Department of Radiology, Shandong Cancer Hospital and Institute, Shandong First Medical University and Shandong Academy of Medical Sciences, Jinan, China; ^2^ Shandong Provincial Key Laboratory of Radiation Oncology, Shandong Cancer Hospital and Institute, Shandong First Medical University and Shandong Academy of Medical Sciences, Jinan, China; ^3^ Shandong Cancer Hospital, Cheeloo College of Medicine, Shandong University, Jinan, China; ^4^ Department of PET/CT Center, Shandong Cancer Hospital and Institute, Shandong First Medical University and Shandong Academy of Medical Sciences, Jinan, China; ^5^ Department of Pathology, Shandong Cancer Hospital and Institute, Shandong First Medical University and Shandong Academy of Medical Sciences, Jinan, China

**Keywords:** radiation-induced lung damage, fibroblast activation protein, macrophage, fibroblasts, positron emission tomography

## Abstract

In this study, we explore the diagnostic value of a novel PET/CT imaging tracer that specifically targets fibroblast activation protein (FAP), ^18^F-NOTA-FAPI, in a radiation induced lung damage (RILD) rat model. High focal radiation (40, 60, or 90 Gy) was administered to a 5-mm diameter area of the right lung in Wistar rats for evaluation of RILD induction. Lung tissues exposed to 90 Gy radiation were scanned with ^18^F-NOTA-FAPI PET/CT and with ^18^F-FDG. Dynamic ^18^F-NOTA-FAPI PET/CT scanning was performed on day 42 post-irradiation. After *in vivo* scanning, lung cryosections were prepared for autoradiography, hematoxylin and eosin (HE) and immunohistochemical (IHC) staining. An animal model of RILD was established and validated by histopathological analysis. On ^18^F-NOTA-FAPI PET/CT, RILD was first observed on days 42, 35 and 7 in the 40, 60 and 90 Gy groups, respectively. After treatment with 90 Gy, ^18^F-NOTA-FAPI uptake in an area of RILD emerged on day 7 (0.65 ± 0.05%ID/ml) and reappeared on day 28 (0.81 ± 0.09%ID/ml), remaining stable for 4–6 weeks. Autoradiography and HE staining IHC staining revealed that ^18^F-NOTA-FAPI accumulated mainly in the center of the irradiated area. IHC staining confirmed the presence of FAP+ macrophages in the RILD area, while FAP+ fibroblasts were observed in the peripheral area of irradiated lung tissue. ^18^F-NOTA-FAPI represents a promising radiotracer for *in vivo* imaging of RILD in a dose- and time-dependent manner. Noninvasive imaging of FAP may potentially aiding in the clinical management of radiotherapy patients.

## Introduction

Radiotherapy is a critical component in the treatment of thoracic malignancies, including esophageal cancer, lung cancer and breast cancer (1). However, normal lung tissue inside the radiation field is vulnerable to potential injury. Radiation doses greater than 50 Gy can lead to the development of radiation-induced lung damage (RILD) in the form of acute radiation-induced pneumonitis (RIP) or late occurring radiation induced lung fibrosis (RILF) (2, 3). High numbers of macrophages are detected within the damaged tissue in clinical and preclinical RILD, as these cells are the first responders to organ injury and are crucial for tissue repair and re-establishment of homeostasis (4). Once the repair process is stimulated by inflammatory cell infiltration, fibroblasts also populate the site of injury and interactions between activated macrophages and fibroblasts coordinate tissue repair after injury, with miscommunications potentially resulting in pathological healing and fibrosis (5). Thus, close monitoring of the responses of macrophages and fibroblasts in radiated tissue can inform new strategies for preventing or delaying the progression of lung fibrosis (6).

Fibroblast activation protein (FAP) is a homodimeric membrane-bound serine protease that has intracellular and extracellular soluble truncated forms (7). Its expression was shown to facilitate the ability of macrophages to migrate through the collagen networks found in the dermis and in the tumor microenvironment, similar to the mechanism demonstrated for FAP-expressing (FAP+) fibroblasts (8). FAP+ fibroblasts are selectively induced in areas of ongoing tissue remodeling, including sites of wound healing (9), fibrosis (10–12), the solid tumor microenvironment (13, 14), and rheumatoid arthritis 15). The basal expression level of FAP in healthy human tissue is considered very low, whereas in mice, detectable levels of FAP expression were shown to be highest in the uterus, pancreas, submaxillary gland, and skin (16). Radiolabeled FAP inhibitors (FAPIs) have been developed for noninvasive imaging of FAP expression and characterized by many groups, exhibiting rapid distribution at the target site and minimal uptake in normal organs (14, 17). In relation to fibrosis, however, FAP remains a relatively understudied protein, and its role in the pathogenesis of this condition is unknown. In the present study, we investigated the feasibility of using a novel FAP-based positron emission tomography (PET)/computed tomography (CT) tracer, ^18^F-NOTA-FAPI, to monitor the injury status of lung tissue following radiation and to define the role of FAP in the development of RILD *in vivo*.

## Methods

### Rats

Male Wistar rats, 6 weeks of age, were obtained from Beijing Vital River Laboratory Animal Technology Co., Ltd. and housed in a specific pathogen-free, temperature and humidity-controlled environment with food and water in their cages. The rats were housed two per cage and allowed to acclimate for 1 week after shipping prior to treatment. All studies involving the use of rats were approved by the Shandong Cancer Hospital and Institute.

### Rat Model

To confirm the feasibility of the proposed experiments, 24 male Wistar rats were randomly assigned to one of four radiation treatment groups (3 rats/group): sham, 40 Gy, 60 Gy, and 90 Gy. Rats were anesthetized with 2.5% pentobarbital administered intraperitoneally and secured onto a four-axis robotic positioning table of a small animal radiation research platform (SARRP; Xstrahl®, Surrey, UK). A high-resolution, treatment planning CT scan was performed, and CT images were reconstructed with an isotropic voxel size of 0.15 mm. The CT image can show the cross section, coronal plane and sagittal plane at the same time, to maximize the survival quality of rats, taking the maximum area of the lung on the coronal plane as the standard, and taking the right lung 2/3 outside the intersection of the outermost edge of the two lungs and the midline of the body as the radiotherapy target area.The diameter of the target area is 5 mm, and 16-18 layers are drawn continuously. The isocenter for radiotherapy planning was positioned in the right lung on coronal, axial, and sagittal slices (**Supplementary Figure 1**). Other parameters such as the target dose according to the dose function, the precise coordinate point and the beam exit time were calculated using MuriPlan software. An arc radiation field was irradiated by a 5 mm×5 mm square field filtered by 0.15 mm Cu in the beam tube. After treatment, the rats were examined weekly by ^18^F-NOTA-FAPI PET/CT scanning to confirm the presence of RILD. RIP/RILF is defined when the uptake of ^18^F-NOTA-FAPI in the irradiated lung is higher on PET/CT than in the contralateral normal lung tissue. All rats that received the 90 Gy radiation treatment developed acute RIP and late occurring RILF based on ^18^F-NOTA-FAPI PET/CT and pathologic evaluations, consistent with previously published observations (18, 19).

**Figure 1:**
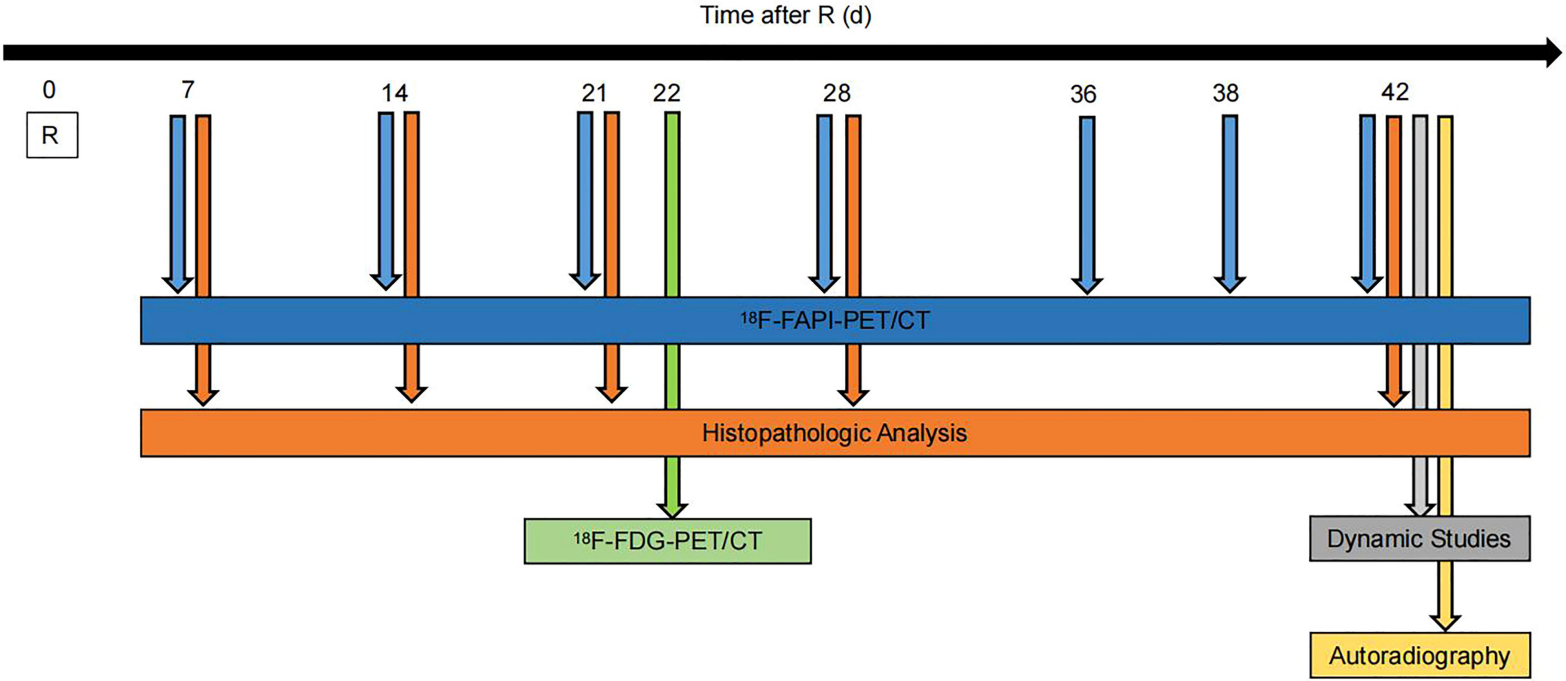
Experimental timeline. Wistar rats were given 90Gy radiation on day 0. Static ^18^F-NOTA-FAPI PET/CT scans were performed at 7, 14, 21, 28, 36, 38 and 42 days after radiation, dynamic ^18^F-NOTA-FAPI-PET/CT scans were performed at 42 days after radiation, and static ^18^F-FDG-PET/CT scans were performed at 22 days after radiation. Autoradiography (n = 3) was performed at 42 days after radiation, and pathological examination was performed at 7, 14, 21, 28 and 42 days after radiation.

In subsequent experiments, 30 male Wistar rats were irradiated with a single dose of 90 Gy under the guidance of CT, with X-ray energy of 220KV and tube current of 13 mA. All experiments were repeated twice, and all findings were similar across all experiments (**Figure 1**).

### Micro PET/CT Imaging

Micro PET imaging was performed using a small-animal Inveon PET/CT scanner (IRIS PET/CT, Inviscan, Germany). Before ^18^F-fluorodeoxyglucose (FDG) PET/CT scanning, the rats were fasted for at least 6 h. The rats were anesthetized with 1.5%–2% isoflurane in a 0.4 L/min flow of air. Static PET/CT images (10 min) were acquired 1 h after injection of ^18^F-NOTA-FAPI (7–9 MBq; days 7, 14, 21, 28, 36, 38, 42 after radiation treatment) or ^18^F-FDG (8–10 MBq; day 22 after radiation treatment). Dynamic PET scanning was performed with ^18^F-NOTA-FAPI (day 42 after radiation treatment) for 90 min.

Images were reconstructed by software that uses a 3-dimensional ordered-subsets expectation maximum algorithm based on the Monte Carlo system model. Image was analyzed using OsiriX MD 7.0 (Pixmeo, Switzerland). Regions of interest were drawn corresponding to the radiation region and a region of contralateral normal lung tissue. The analyzed results were corrected with a decay curve, and signal intensities were recorded as percentage injected dose per milliliter of tissue (%ID/ml). There were 12 frames for a total scan time of 60 minute.

### Autoradiography

To assess the specificity of ^18^F-NOTA-FAPI accumulation and to confirm that uptake of ^18^F-NOTA-FAPI in the area of RILD was due to saturable binding to FAP, a group of treated rats (n=3, day 42 after 90 Gy radiation treatment) were injected with ^18^F-NOTA-FAPI and killed 1 h later. Serial short-axis cryosections 50-µm thick were prepared from the harvested lungs, and consecutive sections were used for autoradiography as previously described (20, 21).

### Histological Analysis

Hematoxylin and eosin (HE) staining (days 7, 14, 21, 28, 42 after radiation treatment) was used to determine the location and extent of areas of RILD, while Masson’s trichrome staining was used to assess overall collagen deposition. IHC stains were carried in the Leica BOND-MAX fully automated staining platform (Leica Biosystems Inc.), the sections were incubated with primary antibodies targeting FAP-alpha (1:100 dilution, ab53066; Abcam) for 20 min and detection was done using Bond Polymer Refine Detection kit with 3,3’-diaminobenzidine (DAB) visualization and Hematoxylin counterstain (DS9800). FAP-alpha was stained with HRP-conjugated secondary antibodies. Full-specimen images were captured using Axio Scan.Z1 (Zeiss).

### Statistical Analyses

Statistical comparisons were performed using the two-tailed Mann-Whitney U test (GraphPad Software, Inc., San Diego, CA). Differences for which the P value was 0.05 or less were considered to be significant.

## Results

### Responses of Rat Normal Lung to Different Doses of Irradiation

**Figure 2:**
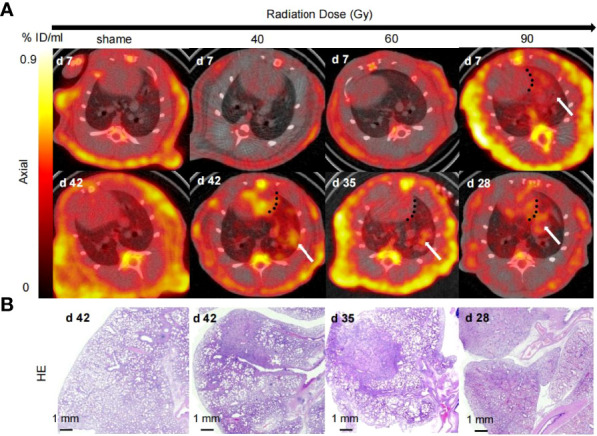
^18^F-NOTA-FAPI PET/CT and pathologic evaluations of rat lung tissues with different radiation doses. **(A)** Micro-CT imaging of FAPI uptake in irradiated rat lung tissue. The black dotted line separates the uptake of tracer in the lung from that in the heart, and the white arrow indicates the presence of RILD in the lung tissue after radiation. Rats in the 40 Gy and 60 Gy groups showed no RIP and began to show RILF on days 42 and 35 after radiation, respectively. In contrast, rats in 90 Gy group showed ^18^F-NOTA-FAPI uptake in areas of RIP on day 7, with reappearance of tracer uptake in the irradiated area on day 28 after radiation. **(B)** Representative micrographs of HE-stained lung sections from the 40 Gy, 60 Gy, and 90 Gy groups at days 42, 35, and 28, respectively, showed that the radiation damage was confined to a small area.

To obtain an initial estimated dose–response curve for lung injury produced by radiation of different doses, areas of normal rat lung were irradiated with three different doses using a 5-mm collimator. In this feasibility study, no RIP was observed in the 40 Gy and 60 Gy groups. However, the rats in the 40 Gy and 60 Gy groups did show RILF on microPET/CT as well as ^18^F-NOTA-FAPI uptake in week 6 (0.76 ± 0.02%ID/ml) and week 5 (0.92 ± 0.06%ID/ml) after radiation treatment (**Figure 2A**). In rats of the 90 Gy group, ^18^F-NOTA-FAPI uptake in areas of RIP emerged on day 7 (0.65 ± 0.05%ID/ml) and reappeared in week 4 after radiation treatment (0.81 ± 0.09%ID/ml). Additionally, in these rats, obvious RILD lesions were observed on micro-PET/CT, and histologic evaluation confirmed the presence of pathological changes associated with RILF in the irradiated lung tissue (**Figure 2B**).

### Rapid Biodistribution and Accumulation of ^18^F-NOTA-FAPI in RILF

**Figure 3:**
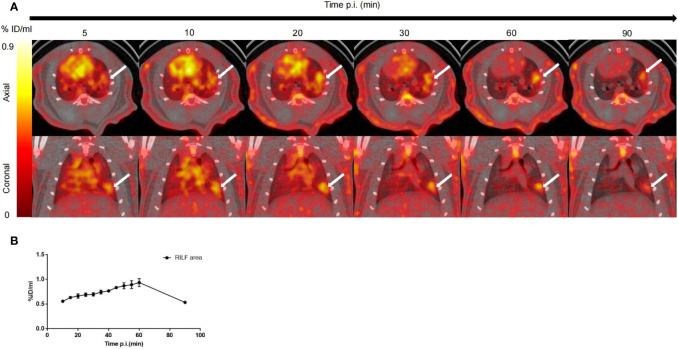
*In vivo* dynamic imaging of ^18^F-NOTA-FAPI uptake in irradiated rat lung tissue. **(A)** Serial PET/CT images (axial and coronal sections) from 90 min of dynamic scanning. White arrows indicate area of RILF. **(B)** Corresponding time–activity curves for fibrosis in lung tissue (average and SD, n=3). p.i. = post-injection.

A series of dynamic images (axial and coronal sections) collected from 5–90 min after injection of ^18^F-NOTA-FAPI in the irradiated lung are presented in **Figure 3A** (day 42 after radiation treatment with 90 Gy). Dynamic measurements over the course of the 90 min post-injection period revealed fast biodistribution and specific tracer uptake in the site of RILF *in vivo* (**Figure 3B**), and FAPI demonstrated the highest uptake level in the injured lung tissue at 60 min after injection (0.93 ± 0.09%ID/ml), followed by a marker decrease within 90 min (0.53 ± 0.01%ID/ml).

### Dynamic Uptake of ^18^F-NOTA-FAPI in RILD

**Figure 4:**
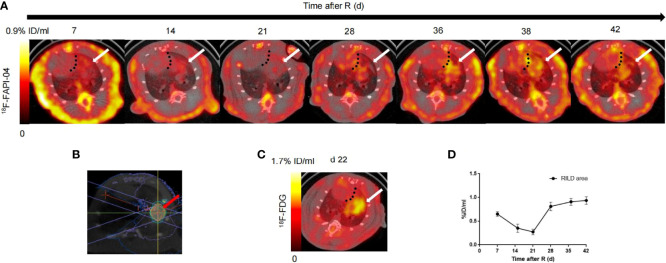
*In vivo* imaging of ^18^F-NOTA-FAPI uptake in longitudinal study. **(A)** Static PET/CT matched axial slices from the same rat subjected to radiation and scanned 1 h after injection of ^18^F-FAPI on days 7, 15, 21, 28, 36, 38 and 42 after radiation treatment. Dashed lines separate tracer uptake in lung from uptake in heart, and white arrows show representative regions of interest (2-dimensional) drawn over area of RILD. **(B)** On the cross section, a schematic diagram of the radiation target (red arrow) of the right lung of rats, an arc field were used to irradiate the lung in a targeted way with a homogeneous dose. **(C)** Static PET/CT matched axial slices in the same rat scanned 1 h after injection of ^18^F-FDG on day 22 after radiation treatment. **(D)** Corresponding time–activity curves for RILD from day 7 to day 42 after radiation (average and SD, n = 3).

**Figure 5:**
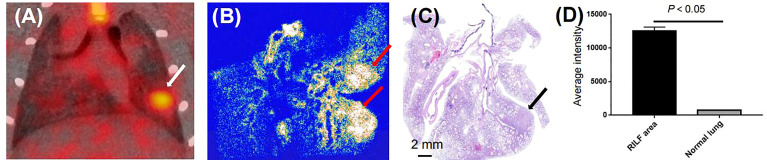
Binding specificity test. **(A)** Serial coronal sections of PET/CT image from 60-min static scan on day 42 after radiation treatment (white arrow). **(B)** Autoradiographs of irradiated lungs show increased ^18^F-NOTA-FAPI uptake in area of RILF at day 42 after radiation treatment (red arrow). **(C)** HE-stained parallel section showed acute RILF (black arrow). **(D)** Comparison of average intensities on autoradiographic imaging between areas of RILF and normal lung (n = 3).

Based on our initial results showing the feasibility of observing differences in RILD severity by ^18^F-NOTA-FAPI-PET/CT imaging in the rat model, we next investigated the potential value of ^18^F-NOTA-FAPI-PET/CT scanning for identifying severe cases of RILD. *In vivo* longitudinal PET/CT images (cross-sections) of a representative rat subjected to damaging lung irradiation are shown in **Figure 4A**. Differential ^18^F-NOTA-FAPI uptake in the area of RILD compared with the normal lung emerged on day 7 (0.26 ± 0.01%ID/ml), and the uptake was significantly elevated in the irradiated area (0.65 ± 0.05%ID/ml, P<0.01), suggesting the emergence of RIP in the lung. The area of RILD was the same as that of rats receiving radiotherapy (**Figure 4B**). Interestingly, the uptake decreased to the background level at 2–3 weeks after irradiation (2 weeks: 0.35 ± 0.09%ID/ml, 3 weeks: 0.27 ± 0.06%ID/ml) before a second increase was observed at 4 week and a stable period from 4–6 weeks (4 weeks: 0.81 ± 0.09%ID/ml, 5 weeks: 0.90 ± 0.07%ID/ml, 6 weeks:0.93 ± 0.09%ID/ml; **Figure 4C**), suggesting the development of RILF. The exact location of ^18^F-NOTA-FAPI uptake within the area of RILF was further verified by autoradiography. In autoradiographic images, increased ^18^F-NOTA-FAPI uptake was observed predominantly in the injured lung area, while no significant tracer uptake was observed in contralateral normal lung tissue (P<0.05; **Figure 5**). Intense ^18^F-NOTA-FAPI uptake observed in the same irradiated area that was identified on ^18^F-FDG PET/CT scans (1.93 ± 0.17%ID/ml; day 22 after radiation treatment; **Figure 4D**).

### Involvement of FAP in the Pathogenesis of RILD

**Figure 6:**
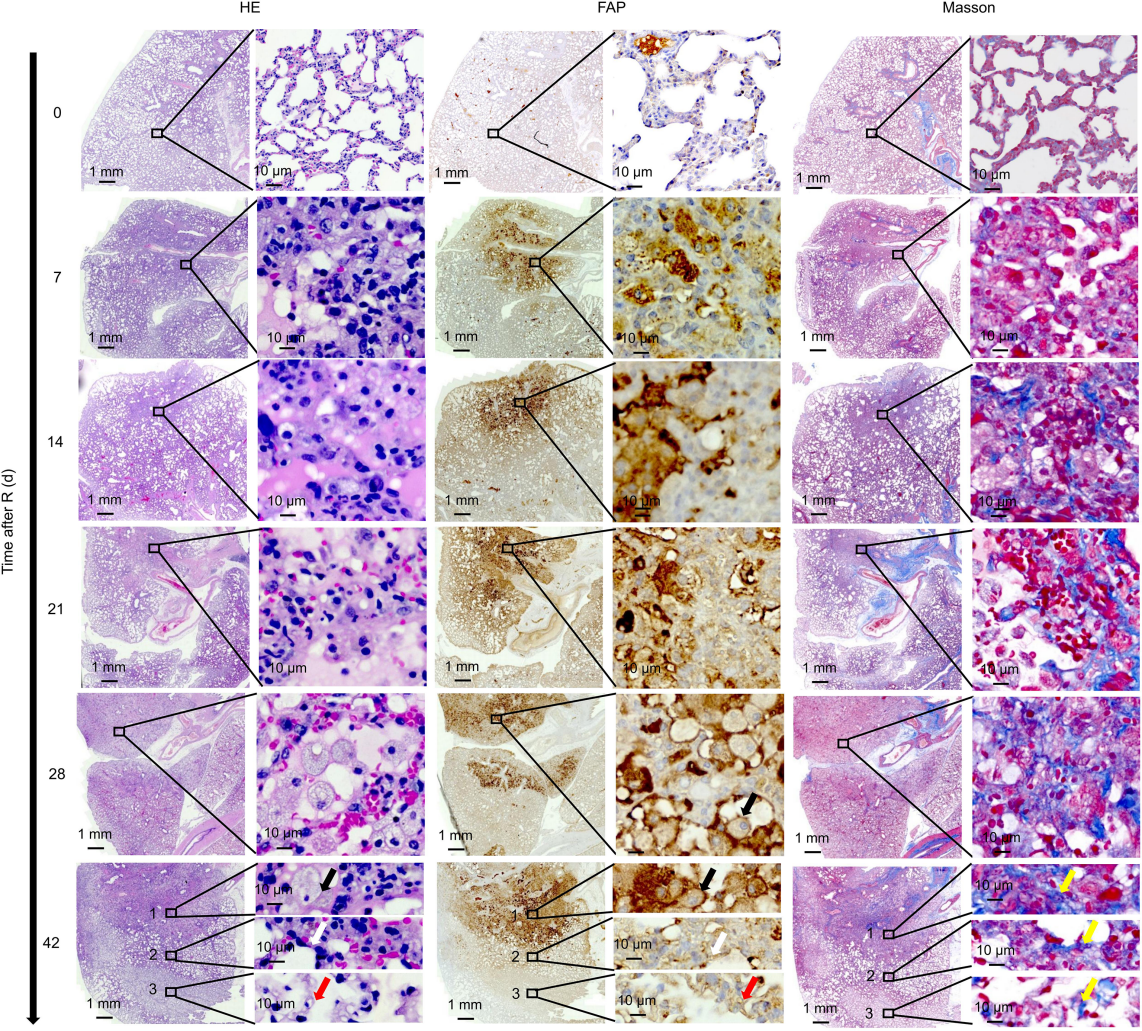
Correlation between ^18^F-NOTA-FAPI uptake and FAP expression. On day 7 after irradiation, foamy macrophages appeared in the damaged lung area, and large numbers of foamy macrophages and fibroblasts reappeared at day 28, remaining until day 42. IHC staining showed abundant FAP expression in the activated macrophages. Slight collagen deposition was detected on day 7, and the amount of collagen gradually increased thereafter based on Masson’s trichrome staining. On day 42 after radiation, infiltration of a large number of FAP+ macrophages were observed (black arrows) along with collagen deposition at the center of the irradiated area. At the border of the irradiated area, thickening of the alveolar walls and a decrease in alveolar air space was observed, leading to disappearance of the alveolar structure (white arrows). At the remote area distant from the irradiated lung tissue, a large number of FAP+ fibroblasts gathered. From the center of the irradiated area to the remote area distant from the irradiated lung tissue, the number of collagens gradually decreased (yellow arrows).

On HE stained sections of the irradiated lung tissue, the radiation damage was confined to a small circular area (**Figure 6**). On day 7 after irradiation, significant cell injury was observed in the irradiated area of the rat lungs, with foamy macrophages present both within and around the scarred area. Many foamy macrophages and fibroblasts were present by day 28 and remained until day 42. IHC staining showed abundant FAP expression in activated macrophages in the injured lung area. Masson’s trichrome staining revealed slight collagen deposition in the injured lung area on day 7 with the amount of collagen deposition gradually increasing thereafter.

On day 42 after radiation, we observed a very interesting “delamination” phenomenon in the RILF area (**Figure 6**). At the center of the irradiated area, we observed a large number of infiltrating FAP+ macrophages and collagen deposition. However, at the border of the irradiated area, we observed thickening of the alveolar walls and a decrease in alveolar air space, leading to the disappearance of the alveolar structure. At the remote area distant from the irradiated area, a large number of FAP+ fibroblasts had gathered and a small amount of collagen had been deposited.

## Discussion

In this comprehensive evaluation of ^18^F-NOTA-FAPI PET/CT for assessing RILD, micro-PET scans showed increases in FAPI uptake in irradiated lung tissue, and the specificity of the PET signal was confirmed by autoradiography and IHC staining, corroborating the high expression of FAP by activated macrophages and fibroblasts. The present study is the first to explore the role of ^18^F-NOTA-FAPI PET/CT in the evaluation and diagnosis of RILD, with pathological examinations used as the gold standard and excluding confounding factors in an animal model. (Avery et al., 2018).

The occurrence of RILD is time, dose and volume dependent (18, 22). Since the emergence of the technical possibility of local lung radiation in rats, a single dose of 90 Gy has been widely used in different studies, which can lead to RILF in lung tissue in 4 weeks (23–27). This preliminary and exploratory study was designed to develop a rat model that would simulate clinical RILD. Exposure to 90 Gy using 5 mm×5 mm beam collimation allowed the development of RILF in a length of time compatible with rat life span. The availability of an image guided SARRP allows high-precision radiation on a millimeter scale (27). Dose volume histograms (DVHs) can be used to ensure target lung irradiation with minimal exposure of other tissues. At several timepoints after radiation, pathological and radiological studies were performed to verify toxicities which offer a good explanation for normal tissue side effects observed in human beings after SBRT, as well as raise a number of questions regarding the time and dose relationships associated with lung tissue response.

The pathogenesis of RILD in patients remains incompletely understood, and greater insight is needed into the events that govern the conversion of what begins as a normal healing process after lung injury into an uncontrolled fibroproliferative response resulting in irreversible scarring, tissue distortion, and progressive decline in lung function. ^18^F-NOTA-FAPI uptake increased at day 7 after radiation and then decreased to the background level by 2–3 weeks before increasing again by 4 weeks and remaining stable thereafter. In clinical settings, RILD can be classified as early (<6 months, i.e., acute RIP) and late (>6 months, i.e., RILF) (28, 29). We also evaluated the histopathologic changes that occurred at several time points to provide a better understanding of the progression of RILD and its consequences. Therefore, ^18^F-NOTA-FAPI PET/CT in the rat model appears to be a good translational model for clinical RILD, allowing for dynamic monitoring of fibroblast activation. This model may be useful for identifying a time window during which fibrosis can still be prevented and the disease course altered.

FAP has been identified in a wide range of cancer types and shows minimal expression in normal tissues (14, 17). Accordingly, several groups have successfully provided proof-of-concept that alpha therapy targeting FAP in the cancer stroma is effective (30, 31). In light of our present findings, however, somewhat heightened caution is needed for such endeavors. For treatment with FAPIs, side effects associated with tracer accumulation in normal tissues or benign lesions may present a major issue, as benign lesions including pulmonary fibrosis, which showed increased FAPI uptake, are common (32, 33). Additionally, many patients with primary lung cancers or other intrathoracic malignancies undergo adjuvant radiation therapy. If future treatment regimens include a pharmacologic FAPI, we need to ensure that patients will not be at increased risk of developing RILD, and a better understanding of the role of FAP in human fibrosing conditions is required.

In this study, ^18^F-FDG PET/CT also contributed to the confirmation of RILD diagnosis. Several groups have investigated FDG-PET imaging as a method for assessing RIP. Guo et al. concluded that ^18^F-FDG imaging may not be able to diagnose aseptic radiation pneumonia in a murine model (34), whereas Abdulla et al. proposed that global lung parenchymal glycolysis and the mean standard uptake value in lung parenchyma may serve as useful biomarkers to quantify lung inflammation on FDG PET/CT following thoracic radiation therapy (35). The imaging targets for the ^18^F-FDG and ^18^F-NOTA-FAPI tracers are different, with ^18^F-FDG revealing glucose metabolism imaging and ^18^F-NOTA-FAPI showing FAP expression. Because ^18^F-NOTA-FAPI provides protease imaging, this method can help guide research into the related mechanisms of RILD that involve FAP.

Although we were able to demonstrate that the process of RILD can be accurately assessed by ^18^F-NOTA-FAPI PET/CT, this study has several limitations. First, we investigated RILD induced by a single high dose of radiation in order to ensure the consistency of the target area. It is possible that RILD caused by radiation delivered in multiple fractions might differ from that produced by a single dose. Although fractionation was not applied in our study, we still assume that our findings provide a valuable reference for understanding ablative dose focal radiation-induced damage to the normal lung. In clinical settings, local control rates of 85–90% are now expected with SBRT when biologically equivalent doses of >100Gy are delivered in several fractionations (36). In addition, based on our study of the time–dose response relationship in radiosensitive rats and previous studies on RILD, we chose 90Gy as a time-effective and well-tolerated ablative dose. A strength of our study was that we used radiotherapy simulation, planning, and delivery to induce targeted RILD, which resembles the clinical situation, and damage was observed in and limited to a local area of the right lung, which represents better accuracy than the previous use of ordinary X-ray irradiation to simulate radiotherapy (37). Moreover, we obtained pathologic confirmation that all irradiated rat lung tissue developed RILD, and the dynamic changes that we observed clearly showed that macrophages and fibroblasts play important roles in the resulting lung fibrosis. More research should be carried out to elucidate the cellular and molecular mechanisms involved.

## Conclusion

RIP and RILF remain dose-limiting forms of radiation-induced lung toxicity with relevant impacts on the success of thoracic radiotherapy. In this study, ^18^F-NOTA-FAPI PET/CT imaging specifically detected inflamed lung tissue containing macrophages expressing FAP in a rat model of RILD, and the findings obtained using this novel tracer can increase our understanding of the dynamic sequential events that occur after radiation of normal lung tissues. Moreover, imaging of FAP expression will be helpful in future attempts to mediate these reactions to reduce the side effects of radiation therapy.

## Data Availability Statement

The original contributions presented in the study are included in the article/**Supplementary Material**. Further inquiries can be directed to the corresponding authors.

## Ethics Statement

The animal study was reviewed and approved by Shandong Cancer Hospital and Institute.

## Author Contributions

SY and YW conceived of the study and participated in its designed; XQ participated in the experiments and drafted the manuscript; SW was responsible for the preparation of ^18^F-NOTA-FAPI and ^18^F-FDG; XL carried out the radiation in rats; JD was responsible for collecting PET/CT images; KC evaluated PET/CT images; ZM carried out the pathology; JJ carried out the nuclear medicine. All authors have read and approved the final manuscript.

## Funding

This study was partially funded by the Natural Science Foundation of China (NSFC81872475, NSFC82073345), the Jinan Clinical Medicine Science and Technology Innovation Plan (202019060), the Bethune Charitable Foundation (flzh202116), and the Natural Science Foundation of Shandong Province (ZR2021QH008).

## Conflict of Interest

The authors declare that the research was conducted in the absence of any commercial or financial relationships that could be construed as a potential conflict of interest.

## Publisher’s Note

All claims expressed in this article are solely those of the authors and do not necessarily represent those of their affiliated organizations, or those of the publisher, the editors and the reviewers. Any product that may be evaluated in this article, or claim that may be made by its manufacturer, is not guaranteed or endorsed by the publisher.
