# Supplementary figures and images for: Diagnostic Value of 18F-NOTA-FAPI PET/CT in a Rat Model of Radiation-Induced Lung Damage
Source: Front Oncol. 2022 Jun 2;12:879281. doi: 10.3389/fonc.2022.879281 (PMC9201039; doi:10.3389/fonc.2022.879281)

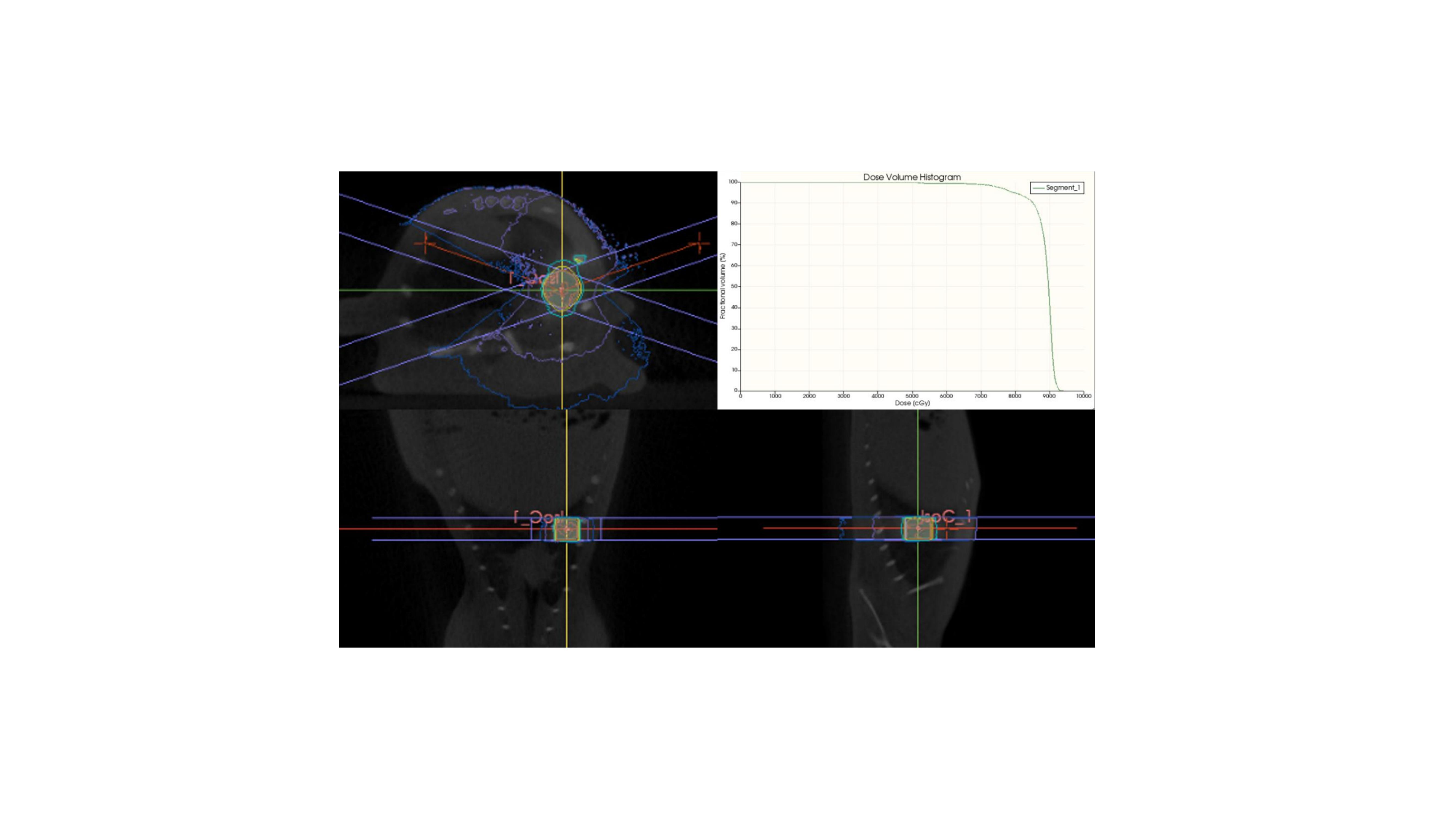

Supplement: Supplementary file 1 [file Image_1.jpeg]
